# Dr. B. T. Whitney

**Published:** 1872-04

**Authors:** 


### OBITUARY.

---

Dr. B. T. Whitney, well known from his connection with the Vulcanizer manufacture, died in Buffalo, N. Y., Jan. 18, 1872, aged 59 years.

"His influence for good was felt in whatever circle he moved, whether professional, religious or social. He was prominent and indefatigable in all movements looking towards the progress of dental science in his State, and considered it a duty to be present at all meetings held for that object within any reasonable distance, To his efforts especially are the dentists of New York indebted for the present State law relating to dentistry.

He successively held the office of President of the Buffalo Dental Association, the Dental Association of Western New York, the Eighth District Dental Society, and the Dental Society of the State of New York, all of which he very ably filled.

Dr. Whitney, after he relinquished his practice, retained his connection with his medical brethren, and always evinced a warm interest in matters relating to general medicine by being present at their meetings. He was a member of the Erie County Medical Society.

In his intercourse with his fellow men he was very genial and friendly, and was governed by the strictest principles of honesty and integrity. In short he was everywhere esteemed as the true type of a christian gentleman."

